# Significant neuronal soma volume deficit in the limbic system in subjects with 15q11.2-q13 duplications

**DOI:** 10.1186/s40478-015-0241-z

**Published:** 2015-10-13

**Authors:** Jerzy Wegiel, Michael Flory, N. Carolyn Schanen, Edwin H. Cook, Krzysztof Nowicki, Izabela Kuchna, Humi Imaki, Shuang Yong Ma, Jarek Wegiel, Eric London, Manuel F. Casanova, Thomas Wisniewski, W. Ted Brown

**Affiliations:** Department of Developmental Neurobiology, NYS Institute for Basic Research in Developmental Disabilities, 1050 Forest Hill Road, Staten Island, NY 10314 USA; Infant Development, NYS Institute for Basic Research in Developmental Disabilities, 1050 Forest Hill Road, Staten Island, NY 10314 USA; Department of Psychiatry, Nemours Biomedical Research, duPont Hospital for Children, Wilmington, DE USA; Department of Psychiatry, University of Illinois at Chicago, Chicago, IL USA; Psychology, New York State Institute for Basic Research in Developmental Disabilities, Staten Island, NY USA; Departments of Pediatrics and Biomedical Sciences, University of South Carolina School of Medicine, Greenville Health System, Greenville, SC USA; Departments of Neurology, Pathology, and Psychiatry, NYU Langone Medical Center, New York, NY USA; Human Genetics, New York State Institute for Basic Research in Developmental Disabilities, Staten Island, Staten Island, NY USA

**Keywords:** Duplications 15q11.2-q13, Autism, Epilepsy, SUDEP, Intellectual disability, Neuropathology

## Abstract

**Introduction:**

Autism is diagnosed in numerous genetic and genomic developmental disorders associated with an overlap in high-risk genes and loci that underlie intellectual disability (ID) and epilepsy. The aim of this stereological study of neuronal soma volume in 25 brain structures and their subdivisions in eight individuals 9 to 26 years of age who were diagnosed with chromosome 15q11.2-13.1 duplication syndrome [dup(15)], autism, ID and epilepsy; eight age-matched subjects diagnosed with autism of unknown etiology (idiopathic autism) and seven control individuals was to establish whether defects of neuronal soma growth are a common denominator of developmental pathology in idiopathic and syndromic autism and how genetic modifications alter the trajectory of neuronal soma growth in dup(15) autism.

**Results:**

Application of the Nucleator software to estimate neuronal size revealed significant neuronal soma volume deficits in 11 of 25 structures and their subregions (44 %) in subjects diagnosed with dup(15) autism, including consistent neuronal soma volume deficits in the limbic system (sectors CA2, 3 and 4 in Ammon’s horn, the second and third layers of the entorhinal cortex and in the amygdala), as well as in the thalamus, nucleus accumbens, external globus pallidus, and Ch3 nucleus in the magnocellular basal complex, and in the inferior olive in the brainstem. The second feature distinguishing dup(15) autism was persistent neuronal soma deficits in adolescents and young adults, whereas in idiopathic autism, neuronal volume deficit is most prominent in 4- to 8-year-old children but affects only a few brain regions in older subjects.

**Conclusions:**

This study demonstrates that alterations in the trajectory of neuronal growth throughout the lifespan are a core pathological features of idiopathic and syndromic autism. However, dup(15) causes persistent neuronal volume deficits in adolescence and adulthood, with prominent neuronal growth deficits in all major compartments of the limbic system. The more severe neuronal nuclear and cytoplasic volume deficits in syndromic autism found in this study and the more severe focal developmental defects in the limbic system in dup(15) previously reported in this cohort may contribute to the high prevalence of early onset intractable epilepsy and sudden unexpected death in epilepsy.

## Introduction

Autism is the most severe form of the developmental disabilities known as autism spectrum disorders (ASDs), which are diagnosed behaviorally as delayed or disrupted development of social and communication skills, restricted interests and stereotypical patterns of behavior and activities, detected prior to 3 years of age [1]. In the majority of individuals diagnosed with autism, the etiology of the disorder is not known (idiopathic autism) [2]. In past decade genetic etiology was identified in about 10 % of individuals with ASD [3, 4], but recently applied new technologies have both increased the identification of putative autism genes [5–7] and raised to approximately 25 % the percentage of children for whom an autism-related genetic change can be identified [8].

The identification of 103 disease genes and 44 recurrent genomic imbalances associated with ASD reflects the etiological heterogeneity underlying these disorders [9]. ASD is among the clinical phenotypes of common genetic disorders, including maternal 15q11-q13 duplications, fragile X syndrome (*FMR1*), Phelan-McDermid syndrome (22q13 deletion syndrome/*SHANK3* mutations), Rett syndrome (*MECP2* duplication syndrome), tuberous sclerosis (TSC1, TSC2), Timothy syndrome (CACNA1C) and cortical dysplasia-focal epilepsy syndrome (CNTNAP2) [9].

### Autism in 15q11-q13 duplication

Maternally derived 15q11-q13 duplications [dup(15)] involving the imprinted Prader-Willi/Angelman region are detected in 1–3 % of all ASD cases [10–12]. Recent studies indicate that 15q duplications (combined interstitial and isodicentric 15q duplications) observed in 1 in 500 cases [13] are the second most prevalent genomic aberration associated with autism, after fragile X syndrome, but penetrance for ASD is much higher in dup 15q11-q13 syndrome [12, 14] than in fragile X syndrome [15–17]. Duplications range from 4 to 12 Mb and may occur either through generation of a supernumerary isodicentric chromosome 15 [idic(15)] or as an interstitial duplication. Array-based comparative genomic hybridization using single-nucleotide polymorphism arrays allowed sub-classification of idic(15) samples by virtue of the lengths of the duplicated region [18].

Most of the reported chromosome 15 duplications are maternally derived. Larger supernumerary idic(15) chromosomes, which include the 15q11-q13 region, are often associated with intellectual disability (ID), seizures, autistic behavior, hypotonia and hyperactivity [19]. A recent study revealed that maternally derived or inherited duplications of the region between BP2 and BP3 were sufficient to produce a phenotype on the autism spectrum in all nine maternal duplication subjects tested [20]. Less common duplications of paternal origin have been reported in clinically asymptomatic individuals [10, 21–24], individuals with clinical symptoms but without the ASD phenotype [20, 23, 25] and subjects with clinical alterations and ASD phenotypes [20, 26]. In several studies of dup(15) cohorts, autism was diagnosed without using standardized measures of autism [27–29], but in the majority of studies, standardized criteria were applied [10, 21, 22, 30–36]. Recent reports reveal that 81 % (44/54 cases) with idic(15) met the DMS-IV criteria for autism, and 92 % (50/54) for ASD [12, 14].

### Overlap between ASD, ID and epilepsy in genetic and neuropathological studies

One of the most striking observations from genetic studies is the considerable overlap in high-risk genes and loci in ASD, ID, and epilepsy [37], reflected in the association of ASD with ID in approximately 70 % of cases, and with epilepsy in 33 % [38]. Genetic and neuropathological studies demonstrate that numerous causative factors act through a similar pathophysiological mechanism, and autism appears to be a final common pathway of behavioral expression for many disorders [39–41]. Autism neuropathology provides evidence of a broad spectrum of developmental abnormalities and inter-individual differences; however, the leading hypothesis proposed by Margaret Bauman is that it is similarities that unify the developmental abnormalities in autism [42]. Neuropathological commonalities range from developmental defects of neuronal proliferation and migration and dysplastic changes [43–47] to reduced size of neurons in cortical minicolumns [48–50] and neurons in brain structures related to autism diagnostic behavioral domains [51–56], as well as abnormalities both related and not related directly to ASD [57–61].

### Hypothesis and aims

Detection of neuropathological commonalities in autism of unknown etiology and autism associated with mutations may reveal a type and topography of pathology with a major contribution to the autistic phenotype. Examination of an idiopathic autism cohort with an increased percentage of larger brains revealed significant reduction of neuronal soma volume in almost all subcortical and cortical regions [60]. Detection of neuronal soma volume deficit in dup(15) autism with significantly smaller brains may strengthen the hypothesis that reduced volume of neuronal soma is a core pathological feature of idiopathic and syndromic autism.

Therefore, the goal of this study is to compare patterns of developmental alterations of neuronal soma volume in eight individuals with dup(15) autism, eight individuals with idiopathic autism and seven age-matched controls. Dup(15) was selected for this study of neurodevelopmental commonalities between idiopathic and syndromic autism because of the high prevalence of the autistic phenotype as well as the high prevalence of intellectual deficits and epilepsy in people with dup(15). To provide a global view of developmental alterations in the brains of people with dup(15) autism and idiopathic autism, 25 brain structures and their cytoarchitectonic subdivisions were examined, including several basal ganglia (amygdala, thalamus, five striatum subdivisions: caudate, putamen, globus pallidus internal and external, and nucleus accumbens), limbic system (Ammon’s horn, entorhinal cortex), neurotransmitter system (magnocellular basal complex with nuclei of the cholinergic system and substantia nigra), cerebellum (Purkinje cells and dentate nucleus) and the inferior olive in the brainstem.

The results of the study support the hypothesis that abnormal neuronal size is a common developmental feature of dup(15)autism and autism of unknown etiology. However, the study also revealed dup15/autism–specific differences in the topography and trajectory of neuronal growth alterations throughout the lifespan.

## Materials and methods

### Cohort

**Table 1:** Material after application of exclusion and inclusion criteria

Group	Case #	Sex	Age (y)	Cause of death	PMI (h)	Hem	Brain weight (g)
Dup(15)	AN14762	M	9	SUDEP	13.6	R	1,130
autism	AN06365	M	10	SUDEP	17.7	R	1,070
	AN09402	M	11	SUDEP	10.5	R	1,540
	AN07740	F	15	SUDEP (suspected)	24.0	L	1,141
	AN09470	F	15	Pneumonia	—	L	1,125
	AN07993	F	22	SUDEP	11.8	L	1,209
	AN05983	M	24	SUDEP	36.3	L	1,200
	AN14829	F	26	SUDEP (suspected)	28.6	R	1,310
	Mean ± SD		16 ± 7		20.3 ± 9.6		1,215 ± 149
Idiopathic autism	HSB4640	M	8	Asthma attack	13.8	R	1,740
	AN01293	M	9	SUDC	3.7	R	1,690
	AN02736	M	15	Pneumonia	2.5	L	1,390
	AN01570	F	18	SUDEP	6.7	L	2,100
	IBR93-01	M	23	Seizure-related	14.0	L	1,610
	AN08166	M	28	Seizure-related	43.0	R	1,580
	NP06-54	M	32	Glioblastoma^a^	—	L	1,260
	AN06420	M	39	Heart failure	13.9	R	1,520
	Mean ± SD		21 ± 11		14.0 ± 13.7		1,611 ± 251
Control	UMB1706	F	8	Cardiac transplant rejection	20.0	R	1,340
	UMB1670	M	14	Asphyxia (hanging)	5.0	R	1,420
	UMB4722	M	14	Traumatic injuries	16.0	R	1,340
	BTB-3960	F	25	Not established	26.0	L	1,520
	UMB4637	F	31	Traumatic injuries	17.0	L	1,354
	IBR291-00	M	32	Heart failure	14.0	R	1,401
	IBR212-98	F	33	Bronchopneumonia	6.0	L	1,260
	Mean ± SD		22 ± 10		14.9 ± 7.4		1,376 ± 81

*PMI* post-mortem interval, *Dup(15)* chromosome 15q11.2-13.1 duplication syndrome, *SUDEP* sudden unexpected death in epilepsy, *SUDC* sudden unexplained death in childhood. Glioblastoma^a^ was found as a single lesion located in the inferior temporal gyrus of the right hemisphere. Neoplastic changes were not detected in the left hemisphere examined morphometrically

Originally, 33 brains, including 11 brains of individuals diagnosed with dup(15), 13 brains of subjects with autism of unknown etiology and nine control subjects, were assigned to this project. However, application of the clinical and neuropathological exclusion criteria reduced the size of the cohort to 23 age-matched cases including eight dup(15) autism, eight idiopathic autism and seven control cases. The brain of one subject with dup(15) autism and two brains of subjects with idiopathic autism were excluded as a result of pre-mortem hypoxia-ischemia and encephalopathy with numerous foci of recent necrosis (Table 1). The brain of one subject with dup(15) autism and two brains of subjects with idiopathic autism were excluded because partial postmortem dissection of the temporal lobe resulted in loss of several structures. To concentrate this comparative study on properties of the cohort with a double diagnosis of dup(15) and autism, one case with dup(15) but without autism was excluded. Two control cases were excluded, one because of detection of clinically not symptomatic but extensive cerebellar dysplasia during neuropathological examination, and the second, because its age did not match any in the autistic cohorts.

Dup(15) is a rare developmental disorder, and the number of postmortem studies of brains of individuals diagnosed with dup(15) is very limited. Therefore, the age of individuals diagnosed with dup(15), from 9 to 26 years (mean age ± SD; 16 ± 7), was the restraining factor in selection of age-matched control subjects (from 8 to 33 years; 22 ± 10) and subjects with idiopathic autism (from 8 to 39 years; 21 ± 11). The ratio between males and females was 4:4 in the dup(15) autism group, 3:4 in the control group, and 7:1 in the idiopathic autism group.

Sudden and unexplained death in patients with epilepsy (SUDEP) was reported in seven of eight (87 %) cases in the dup(15) cohort. In the idiopathic autism group, the SUDEP was reported in one case, and death was related to epilepsy in two other cases (total of three cases per eight examined; 37 %). In control cases, the cause of death was due to heart or respiratory system failure (three cases), multiple traumatic injuries related to car accident (two cases) or suicide (one case). In one case, the cause of death was not established.

### Chromosome 15 abnormalities in dup(15) cohort

Tetrasomy of the Prader-Willi syndrome/Angelman syndrome–critical region was detected in seven subjects with dup(15). In an 11-year-old boy, a complex tricentric supernumerary chromosome arising from nonallelic homologous recombination between multiple copies of BP3 rendered him hexasomic for the region between the centromere and BP3 [62] (case 2); [63] (case 00–29). Abnormalities were determined as being of maternal origin in all dup(15) cases except for a 22-year-old female with undetermined origin of two extra copies of the 15q11.2-q13.1 region. A more detailed description of all eight individuals diagnosed with dup(15) was published by Wegiel et al. [46] based on reports by Mann et al. [62] and Wang et al. [63].

### Clinical characteristics and diagnosis of autism

**Table 2:** Psychiatric and neurological assessment

Group	Case#	Psychiatric disorders, neurological symptoms	Cognitive assessment	Seizures (age of onset)	Diagnosis of autism
Dup(15) autism	AN14762	Regression in infancy. Hypotonia. Abnormal response to pain and heat	Profound ID (DQ < 20)	Infantile spasms Intractable epilepsy (10 m)	Autism (ADOS-G)
	AN06365	Severe regression at age of 15 months. Hypotonia	Profound ID	Intractable epilepsy (8 m)	Autism (ADI-R)
			(DQ = 22)		
	AN09402	Regression in infancy. Severe hypotonia	Profound ID	Intractable epilepsy (10 m) VNS	PDD-NOS
			(DQ < 20)		
	AN07740	Delay of motor skills. Moderate spastic quadriparesis. Abnormal response to pain and heat	Severe ID	Seizures (11 y)	Autism (ADI-R)
			(DQ = 31)		
	AN09470	Hyperactivity	(—)	No record	Autism (ADI-R)
	AN07993	(—)	(—)	Epilepsy (9 y)	Autism (ADI-R)
	AN05983	Abnormal gait	Profound ID	Intractable epilepsy (7 y). VNS. Callosotomy	Autism (ADI-R)
			(DQ < 20)		
	AN14829	Obsessive compulsive behavior	Moderate ID	Epilepsy (16 y)	Autism (ADI-R)
			(IQ = 36)		
Idiopathic autism	HSB4640	Self-stimulatory behavior	(—)	Epilepsy (8 y)	Atypical autism (ADI-R)
	AN01293	Hypotonia	Moderate ID	No record	Autism (ADI-R)
	AN02736	Hypotonia. Sleep disorder	Moderate ID	Epilepsy (2 y)	Autism (ADI-R)
	AN01570	(—)	(—)	Epilepsy (8 y)	Autism (ADI-R)
	IBR93-01	Hyperactivity. Aggression. Sleep disorder	Moderate ID	Seizure (23 y)	Autism (ADI-R)
			(IQ = 36)		
	AN08166	Bipolar disorder	(—)	Epilepsy	Autism (ADI-R)
	NP06-54	No record	(—)	No record	Autism (ADI-R)
	AN06420	Impulsivity. Aggression	(—)	No record	Autism (ADI-R)

*Dup(15)* chromosome 15q11.2-13.1 duplication syndrome, *ID* intellectual disability, *DQ* development quotient, *ADOS-G* Autism Diagnostic Observation Schedule–Generic, *ADI-R* Autism Diagnostic Interview-Revised, *VNS* vagus nerve stimulator

The source of clinical data was the medical records of the autistic subjects, which consisted of psychological, behavioral, neurological and psychiatric evaluation reports. All of the records were obtained after the subjects’ deaths. The Autism Diagnostic Interview-Revised (ADI-R) was administered to each donor family as a standardized assessment tool to confirm autism diagnosis on a postmortem basis. The ADI-R provides scores of four domains: (a) qualitative abnormalities in reciprocal social interaction; (b) qualitative abnormalities in verbal and nonverbal communication; (c) restricted, repetitive and stereotyped patterns of behavior; and (d) abnormality of development evident at or before 36 months [1].

Of the eight subjects with dup(15), diagnosis of autism was confirmed with the ADI-R in six cases (Table 2). In a 9-year-old boy, autism had been diagnosed with the Autism Diagnostic Observation Schedule–Generic (ADOS-G) before death. In an 11-year-old boy, impairments were consistent with pervasive developmental disorder–not otherwise specified. Average scores on the ADI-R scale, including reciprocal social interactions (score 21 ± 7 in dup(15) autism and 24 ± 7 in idiopathic autism); verbal deficits in communication (20 ± 3 and 17 ± 6, respectively); restricted, repetitive and stereotyped behavior (5 ± 4 and 6 ± 2, respectively); and behavioral alterations evident before 36 months (4 ± 1 in both groups) reveal no significant difference between clinical autism symptoms in the dup(15) autism and in the idiopathic autism cohorts examined postmortem.

In the examined cohort, epilepsy was diagnosed in seven of eight (87 %) individuals diagnosed with dup(15), including intractable early-onset epilepsy reported at the age of 8 months, at 10 months in two children, and at 7 years. In two individuals, vagus nerve stimulator was applied, and in one subject, application of stimulator was followed by callosotomy. In the idiopathic autism group, epilepsy was reported in four of eight cases (50 %), whereas in one case, the first seizure was the cause of death.

In the dup(15) group, intellectual deficit was reported in six cases, including profound deficit in four individuals (67 %), and severe and moderate in two other cases. Records regarding intellectual deficit were available for only three cases of idiopathic autism, all three of them diagnosed with moderate ID.

### Brain tissue preservation

The difference between mean postmortem intervals (PMI, Table 1) in dup(15) autism (20.3 ± 9.6), idiopathic autism (14 ± 13.7) and control group (14.9 ± 7.4) was insignificant. Brains were removed using standard techniques, weighed, and sagittally cut through the corpus callosum and brainstem. The mean brain weights at autopsy were 1,215 (±149) g in the dup(15) group, 1,611 (±251) g in idiopathic autism, and 1,376 (±81) g in control group. The difference between dup(15) autism and idiopathic autism was significant, but differences between control and two autistic groups were insignificant.

One brain hemisphere was fixed in 10 % buffered formalin for more than several months. After approximately three weeks, the brain hemisphere was scanned using MRI. The topography, type and size of developmental abnormalities were determined by postmortem MRI and neuropathological examination [46]. Brain hemispheres were dehydrated in a graded series of increasing ethyl alcohol concentrations for 14 days, infiltrated with polyethylene glycol (PEG) 400 (no. 807 485; Merck, Whitehouse Station, NJ) and stored at 4 °C. Serial 50-μm-thick hemispheric sections were cut, and numbered free-floating sections were stored in 70 % ethyl alcohol. Sections were washed in water for 6–12 h, stained with cresyl violet (CV) and mounted with Acrytol. Neuropathological examination of approximately 140 serial sections with a 1.2-mm distance between sections revealed 2.5 times more developmental defects of neuronal migration in the dup(15) autism than in the idiopathic autism cohort [46].

Tissue acquisition was based on individual tissue transfer agreements between the project’s principal investigator and several tissue banks, including (1) the National Institute of Child Health and Human Development Brain and Tissue Bank for Developmental Disorders at the University of Maryland School of Medicine, MD, (2) the Harvard Brain Tissue Resource Center, McLean Hospital, Belmont, MA, and (3) the Brain and Tissue Bank for Developmental Disabilities and Aging of the New York State Institute for Basic Research (IBR) in Developmental Disabilities. IBR’s Institutional Review Board approved the methods applied in this study.

### Stereological and statistical analysis

To establish the difference between neuronal size in subjects diagnosed with autism caused by dup(15) and autism of unknown etiology, the volume of neuronal soma has been estimated with the nucleator method [64]. To identify the global pattern of developmental abnormalities of neuron growth, 25 brain structures and their subdivisions (Table 3) were examined, including amygdala; thalamus and magno- and parvo-cellular lateral geniculate nucleus; claustrum; four striatum subdivisions, caudate, putamen, external and internal globus pallidus; four subdivisions of the magnocellular basal complex (Ch1, 2, 3 and 4); dorsal and ventral portions of the substantia nigra; four sectors of the cornu Ammonis (CA1, 2, 3 and 4); and two layers in the entorhinal cortex (islands in the second layer and layer III). In the cerebellum, the volumes of Purkinje cells and neurons in the dentate nucleus were estimated. In the brainstem, the size of neurons was established in the inferior olive.

**Table 3:** Stereological parameters and procedures applied to estimate neuronal soma volume

Structures and their subdivisions	Number of sections examined (per case)	Obj.	Grid size (μm)	Test area size (μm)	Mean number ± SD		CE^a^
					Virtual counting spaces (per case)	Neurons measured (per case)	
Amygdala	5	40×	2,000 × 2,000	60 × 60	133 ± 32	170 ± 5	0.01
Thalamus	6	40×	2,000 × 2,000	60 × 60	286 ± 82	219 ± 15	0.01
Entorhinal cortex (EC): Isl	3	100×	400 × 400	50 × 50	69 ± 27	107 ± 4	0.004
EC: L III	3	100×	800 × 800	50 × 50	57 ± 17	106 ± 4	0.004
Ammon’s horn: CA1	3	100×	1,000 × 1,000	50 × 50	41 ± 13	107 ± 4	0.004
CA2	3	100×	400 × 400	50 × 50	30 ± 10	106 ± 3	0.003
CA3	3	100×	400 × 400	50 × 50	44 ± 17	105 ± 3	0.004
CA4	3	100×	600 × 600	50 × 50	37 ± 15	105 ± 4	0.004
Caudate nucleus	4	63×	1,000 × 1,000	80 × 80	197 ± 70	340 ± 78	0.001
Putamen	4	63×	1,000 x 1,000	80 × 80	162 ± 75	329 ± 103	0.001
Globus pallidus	6	63×	500 x 500	60 × 60	663 ± 263	190 ± 97	0.001
Nucleus accumbens	6	63×	500 × 500	80 × 80	306 ± 100	447 ± 115	0.003
Magnocellular LGN	4	40×	500 × 500	100 × 100	211 ± 49	353 ± 33	0.001
Parvocellular LGN	4	40×	500 × 500	100 × 100	211 ± 49	353 ± 33	0.001
Claustrum	5	40×	600 × 600	60 × 60	417 ± 287	276 ± 44	0.01
Substantia nigra	8	63×	300 × 300	80 × 80	392 ± 104	231 ± 97	0.003
Magnocellular basal complex Ch1	4	63x	300 x 300	80 x 80	41 ± 12	87 ± 26	0.007
Ch2	4	63×	300 × 300	80 × 80	64 ± 16	143 ± 35	0.003
Ch3	4	63×	300 x 300	80 × 80	90 ± 39	189 ± 47	0.003
Ch4	4	63×	300 × 300	80 × 80	133 ± 49	240 ± 42	0.002
Purkinje cells	5	40×	1,800 × 1,800	180 × 180	1,118 ± 209	449 ± 28	0.001
Dentate nucleus	4	40×	1,000 × 1,000	180 × 180	178 ± 39	425 ± 28	0.002
Inferior olive	6	40×	600 × 600	80 × 80	110 ± 28	129 ± 23	0.003

*Obj.* objective lens, *CE* average predicted coefficient of error of the measured neurons (Scheaffer), *LGN* lateral geniculate nucleus

Measurements of the volume of neuronal soma were supported with the MicroBrightfield software (Nucleator, Stereo Investigator, version 7.003, MBF Bioscience, Williston, VT, USA). Five points of intersection of the systematic randomly rotated five radii with the cell border were marked. Measurements were performed using a workstation consisting of an Axiophot II (Carl Zeiss, Goettingen, Germany) light microscope with a specimen stage with a three-axis, computer-controlled stepping motor system (Ludl Electronics; Hawthorne, NY, USA); and a CCD color video camera (CX9000 MBF Bioscience). Borderlines of region of interest were determined at low magnification with Plan Apo objectives 1.25x (numerical aperture, N.A., 0.15) or 2.5x (0.075), whereas neuronal soma volume was estimated with objectives 40x (0.75), 63x (0.9) or 100x (1.3).

The number of equidistant sections examined with Nucleator varied from three in structures with relatively uniform cytoarchitecture to eight in structures with multiple subdivisions (substantia nigra). An optical fractionator systematic random sampling scheme was supported with Stereo Investigator (MicroBrightfield Bioscience, Inc., Williston, VT, USA). The number of virtual counting spaces ranged from 30 (CA2) to 663 in the globus pallidus, but for Purkinje cells, the number of counting spaces was increased to 1,118. To reduce a Schaeffer coefficient of error to less than 5 %, the number of examined neurons per region in individual case ranged from 87 in Ch2 in the MBC to 449 for Purkinje cells. Anatomical boundaries of the examined brain structures and their subdivisions were described in Wegiel et al. [60].

This study analyzed the effect that autism has on neuronal soma volume in 25 brain structures and their anatomical subdivisions in age-matched samples of subjects with autism associated with dup(15), subjects with autism of idiopathic origin, and control subjects. Any data points more than 1.5 times the interquartile range below the 25th percentile or an equal amount above the 75th percentile for each structure or substructure were considered outliers and were omitted from analyses. The removed data accounted for approximately 3.4 % of all records. Following the procedure developed in our previous analyses of the effects of potential confounders including PMI in hours, fixation time in days, brain weight in grams, percentage brain weight loss during processing and dehydration, duration of dehydration in days, history of seizures, and SUDEP, analyses were controlled for PMI (log-transformed), days of dehydration, and weight loss during dehydration. Statistical significance of differences in mean neuronal volumes in the dup(15) autism, idiopathic autism, and control groups were computed in general linear mixed models using the mixed procedure in version 13.1 of the Stata statistical package [65].

## Results

**Fig. 1:**
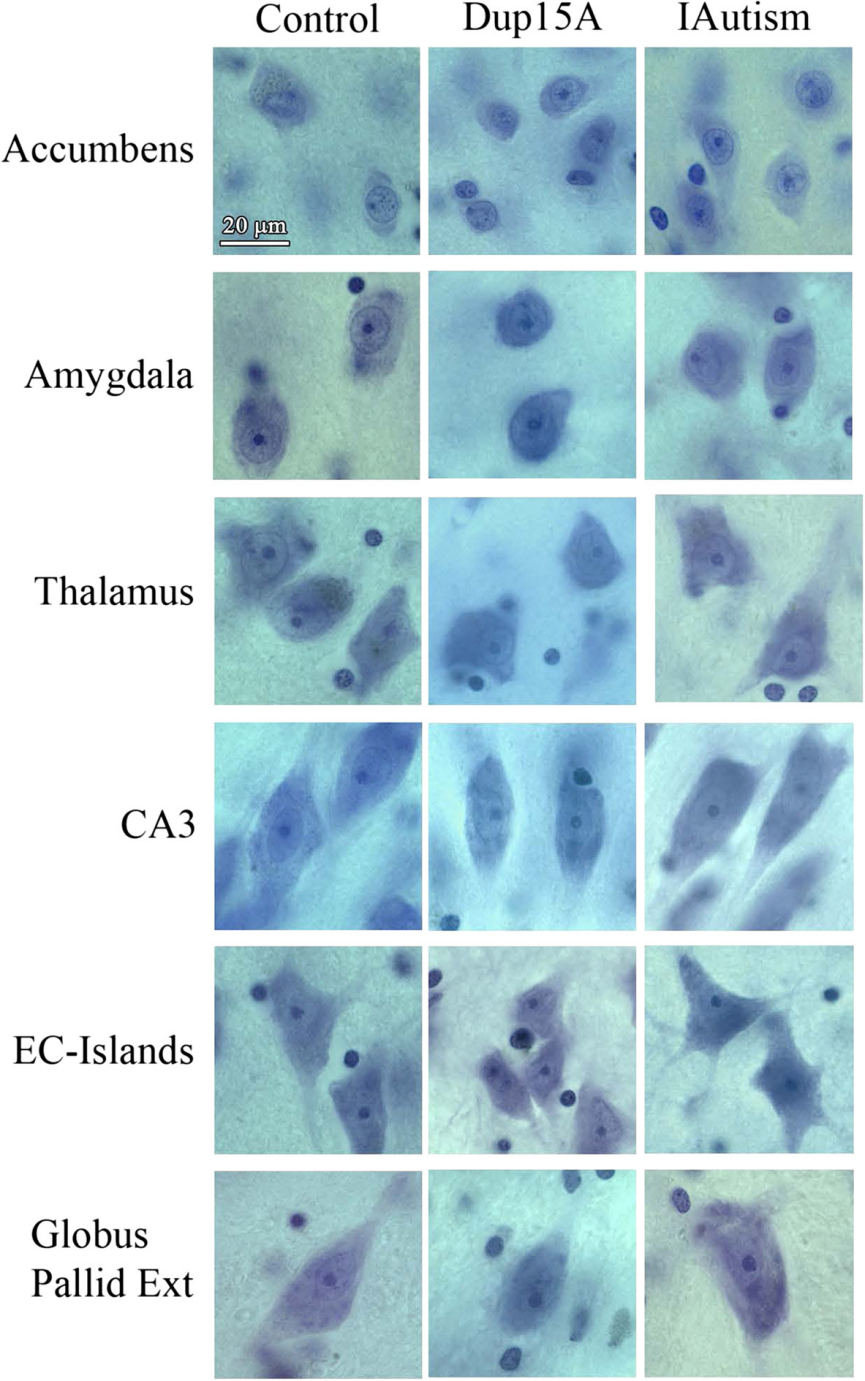
Neuronal size in control, dup(15) autism and idiopathic autism. Panel of micrographs demonstrates smaller neurons in the nucleus accumbens, amygdala (lateral nucleus), thalamus (dorsal medial nucleus), CA3 sector in the Ammon’s horn, the second layer of the entorhinal cortex (islands of stellate neurons; ECIsl) and the external globus pallidus in a 24-year-old male diagnosed with dup(15), compared to a 32-year-old control male and a 23-year-old male diagnosed with idiopathic autism (IAutism). Stereology revealed significant (from 17 % to 23 %) neuronal soma volume deficit in these structures in dup(15) autism. Staining with cresyl violet. Calibration bar, 20 μm

Examination of serial CV-stained brain sections revealed abnormally small neurons mixed with normal size neurons, and fewer large neurons in almost all the examined regions in individuals diagnosed with dup(15) autism from 9 to 26 years of age. Figure 1 demonstrates smaller neurons in the nucleus accumbens, amygdala, thalamus, CA3 sector of the Ammon’s horn, the second layer (islands of stellate neurons) in the entorhinal cortex and the external part of the globus pallidus in a 24-year-old male diagnosed with dup(15) autism. In some regions, especially in the islands of stellate cells in the entorhinal cortex, small neurons were arranged in clusters. Comparison of neurons in these brain structures in teenagers and adults does not reveal smaller neurons in the idiopathic autism than in the control group. However, in subjects with autism of unknown etiology, smaller neurons were a common finding in the claustrum and magnocellular basal complex.

### Neuron soma abnormalities detected with nucleator

**Fig. 2:**
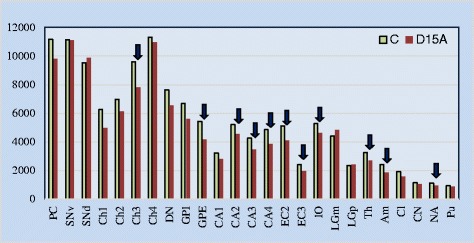
Neuronal soma volume deficit in dup(15) autism. Comparison of neuronal soma volume between dup(15) autism and control subjects revealed a significant neuronal soma volume deficit in affected subjects in 11 brain structures or their subdivisions (marked with arrows) per 25 examined (44 %), but a trend to lower cell soma volume is reflected in the insignificantly lower soma volume in another 11 brain structures in dup(15) autism cases from 9 to 26 years of age

In subjects diagnosed with dup(15) autism, application of nucleator revealed significant neuronal soma volume deficit in 11 of 25 structures and their subregions (44 %), including the amygdala, thalamus, nucleus accumbens, globus pallidus external, and Ch3 nucleus in the magnocellular basal complex; and surprisingly consistent neuronal soma volume deficit in three sectors (CA2, 3, 4) in the Ammon’s horn, in the second and third layers in the entorhinal cortex, and in the inferior olive in the brainstem (Table 4). Significant neuronal soma volume deficit ranged from 13 % to 24 %; 12 % deficit of Purkinje cells’ volume did not reach the significance level. Significantly smaller volume in 11 regions and insignificantly lesser volume of neurons in another 11 regions appears to reflect developmental neuronal alterations in 88 % of the examined structures in dup(15) autism (Fig. 2).

**Table 4:** The difference between the mean volume of neuronal soma in control, dup(15) autism [dup(15)A] and idiopathic autism (iAutism) subjects

Brain structure	Mean volume of neuronal soma ± SD			Difference between groups (% and *p*<)		
	Control	Dup(15)A	iAutism	Control/Dup(15)A	Control/iAutism	iAutism/dup(15)A
Amygdala	2,424 ± 1,157	1,861 ± 1,048	2,327 ± 1,176	−23 % (0.000)		−20 % (0.024)
Thalamus	3,239 ± 1,379	2,678 ± 1,231	3,328 ± 1,430	−17 % (0.003)		
Magnocellular LGN	4,397 ± 2,040	4,821 ± 1,969	4,716 ± 1,960			
Parvocellular LGN	2,338 ± 855	2,395 ± 887	2,514 ± 881		+7 % (0.019)	−5 % (0.016)
Claustrum	1,914 ± 764	1,584 ± 739	1,582 ± 695		−17 % (0.004)	
Caudate nucleus	1,133 ± 393	1,052 ± 366	1,286 ± 380			
Putamen	931 ± 325	875 ± 300	1,022 ± 306			
Nucleus accumbens	1,122 ± 313	919 ± 292	1,089 ± 327	−18 % (0.003)		
G. pallidus ext.	5,438 ± 2,241	4,150 ± 1,931	5,117 ± 2,160	−24 % (0.001)		−19 % (0.048)
G. pallidus int.	6,670 ± 3,011	5,591 ± 2,715	6,069 ± 2,842			
Magnocellular basal complex Ch1	6,252 ± 2,787	4,972 ± 2,894	4,729 ± 2,500		−24 % (0.002)	+5 % (0.009)
Ch2	6,966 ± 2,813	6,117 ± 2,800	5,507 ± 2,777		−21 % (0.007)	
Ch3	9,592 ± 3,619	7,820 ± 3,291	7,122 ± 3,318	−18 % (0.030)	−26 % (0.000)	
Ch4	11,314 ± 4,242	10,956 ± 3,890	9,949 ± 3,871			
Substantia N. Dors.	9,527 ± 4,350	9,864 ± 4,403	9,162 ± 4,286			
Substantia N. Ven.	11,138 ± 4,474	11,099 ± 4,607	10,417 ± 4,900			
Ammon’s horn CA1	3,215 ± 1,241	2,801 ± 1,127	3,211 ± 1,233			
CA2	5,204 ± 1,822	4,536 ± 1,717	5,696 ± 1,965	−13 % (0.014)		
CA3	4,251 ± 1,624	3,450 ± 1,347	4,291 ± 1,596	−19 % (0.000)		−20 % (0.010)
CA4	4,855 ± 1,817	3,857 ± 1,615	5,087 ± 1,825	−20 % (0.001)		−24 % (0.000)
Entorhinal Cortex: LII, Islands	5,116 ± 1,960	4,102 ± 1,723	4,548 ± 1,991	−20 % (0.047)		
EC LIII	2,403 ± 985	1,957 ± 885	2,311 ± 914	−19 % (0.049)		
Purkinje cells	11,164 ± 3,948	9,818 ± 3,747	10,849 ± 3,934		−3 % (0.024)	
Dentate nucleus	7,636 ± 4,414	6,547 ± 3,939	7,793 ± 4,541			
Inferior olive	5,269 ± 1,978	4,610 ± 1,639	5,264 ± 1,883	−13 % (0.023)		

*dup(15)* chromosome 15q11.2-13.1 duplication syndrome, *LGN* lateral geniculate nucleus

The pattern observed in the dup(15) autism group compared with the control group is in striking contrast to the pattern detected in the idiopathic autism group compared with the control group. There was no neuronal volume deficit in the structures affected in the dup(15) group, but significant deficit was detected in the claustrum and three nuclei in the magnocellular basal complex (Ch1, 2 and 3). In idiopathic autism, the deficit in the volume of Purkinje cells was small but statistically significant. Comparison between dup(15) autism and two other groups revealed that in six regions (the amygdala, parvocellular lateral geniculate nucleus, globus pallidus external, in Ch1 in magnocellular basal complex, and CA3 and 4 sectors in the Ammon’s horn), the volume of neuronal soma was less than in controls and idiopathic autism (Table 4).

To determine whether the neuron volume deficit in the autistic group might be due to epilepsy, seven dup(15) cases with seizures were compared with one case without epilepsy. Despite the extremely unbalanced number of subjects in sub-groups, the analyses showed a significantly smaller neuron size in the amygdala (*p* = 0.010) and the CA3 (*p* = 0.018) for those with epilepsy. The same analyses in the idiopathic autism group with four subjects diagnosed with epilepsy and four without epilepsy, showed significantly smaller neurons in all four sectors of the CA in subjects diagnosed with epilepsy (CA1 and 2, *p* = 0.001; CA3, *p* = 0.006, and CA4, *p* = 0.015) than in subjects without epilepsy. Comparison of the dup(15) group vs. the idiopathic autism group controlling for presence of epilepsy revealed essentially unmodified results. For the amygdala the *p* value changes from 0.024 to 0.034; for CA3 it changed from 0.010 to 0.64; for CA4 it changed from 0.001 to 0.002.

### Neuronal soma volume alterations demonstrated by analysis of frequency distribution

**Fig. 3:**
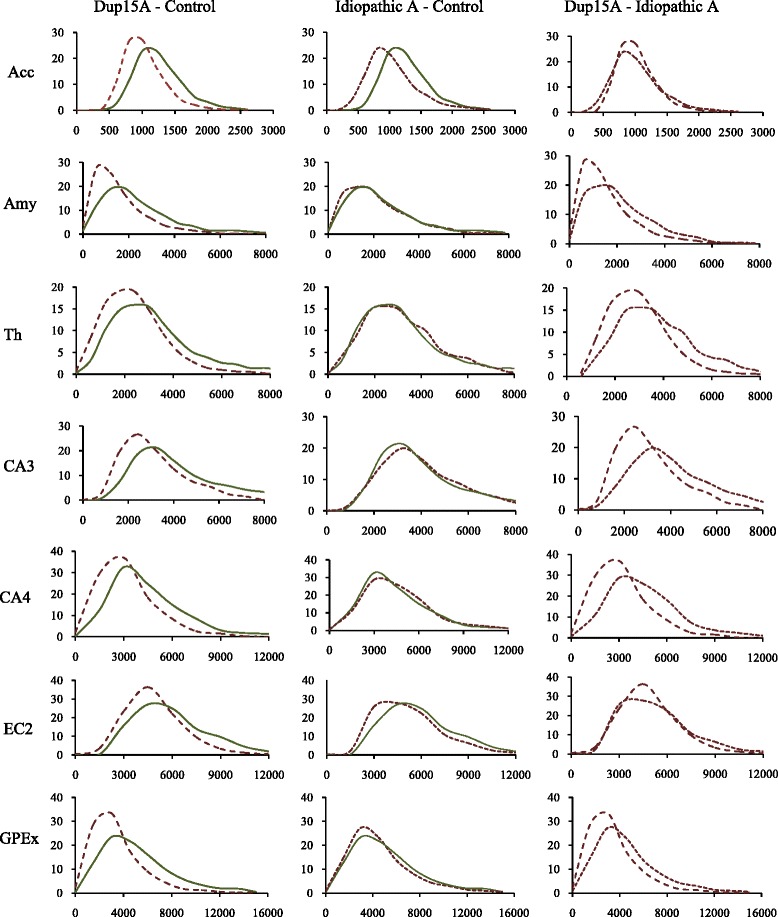
Neuronal soma volume frequency distribution. Comparison of neuronal soma frequency distribution in dup(15) autism group (9–26 years old), idiopathic autism (IA) group (8–39 years old) and control group (8–33 years old). Graphs arranged in order from the smallest to the largest neurons demonstrate a significant (asterisks) shift towards small neurons in the nucleus accumbens (Acc), amygdala (Amy), thalamus (Th), CA3 and CA4 sectors in the Ammon’s horn, second layer in the entorhinal cortex (EC2) and the globus pallidus external (GPEx) in the dup(15) group (segment-line) in comparison to age-matched control subjects (continuous line). However, at this age, the difference in neuronal body volume distribution in these structures is not significant when subjects with idiopathic autism (IA) (dot-line) are compared to control subjects (C). The shift to smaller neurons was significant in the dup(15)/A cohort when compared with IA in the amygdala, CA3 and CA4 sectors, and the globus pallidus external. A similar shift in the thalamus did not reach significance level but is consistent with the trend observed in other structures

Frequency distribution demonstrates a uniform pattern of changes, with an increased percentage of small neurons and a decreased percentage of large neurons in the nucleus accumbens, amygdala, thalamus, CA3 and CA4 sectors in the Ammon’s horn, the islands in the second layer in the entorhinal cortex, and the external part of the globus pallidus in subjects with dup(15) autism (Fig. 3). However, the difference between the idiopathic autism and control groups was undetectable, except in the nucleus accumbens, with a similar shift towards small neurons. Comparison of dup(15) autism with idiopathic autism shows more small neurons and fewer large neurons in dup(15) autism in the amygdala, thalamus, CA3 and 4, and the globus pallidus external.

### Box plots

**Fig. 4:**
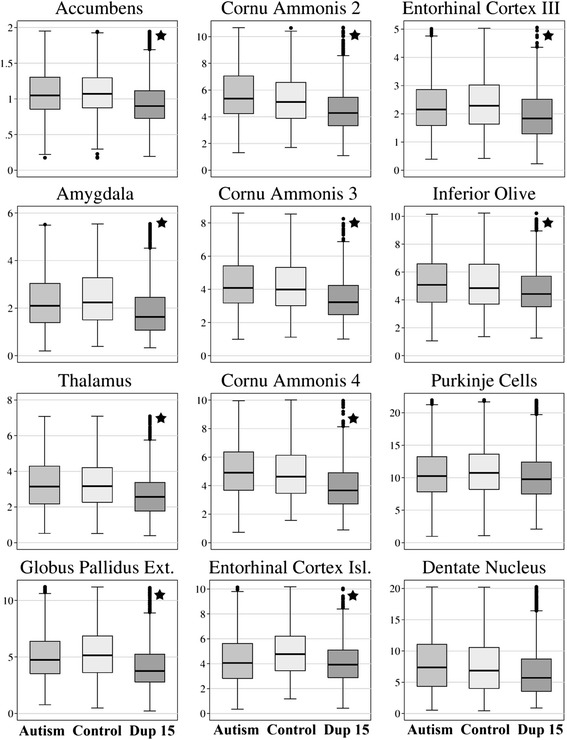
Neuronal soma volume distributions. Box plots illustrate neuronal body volume distributions in 10 structures with significantly smaller neurons (marked with a star) in 8- to 26-year-old autistic individuals with dup(15) compared to age-matched control subjects. Purkinje cells and neurons in the dentate nucleus were also smaller, but the difference did not reach the level of significance. Volume measures on the Y axis correspond to x1000 of μm^3^. The upper and lower boundaries of each box represent the interquartile range (IQR), whereas the whisker above and below each box marks the maximum and minimum values unless any data point lies more than 1.5 times of the IQR above the 75^th^ percentile or 1.5 times below the 25^th^ percentile. The characteristic features of neuron volume distribution in the dup(15) autism group in all 12 structures were lower median values (marked with horizontal line within box), lower range of 50 % of data (box range), and fewer large neurons (much lower maximum values marked by whiskers above the box). A few neurons in range above the 75^th^ percentile are indicated by circles; however, as a result of much lower maximum values outliers occur more often in dup(15) autism

Box plots distinguish dup(15) autism from the idiopathic autism and control groups with (a) lower median neuronal soma volume, (b) lower range of 50 % of cell volume records (box range), and (c) lower percentage of large neurons within the 75^th^ percentile (Fig. 4). Box plots reveal a striking deficit of large neurons in subjects with dup(15) autism, with only a few neurons in the top range of control or idiopathic autism cases.

## Discussion

Our previous study of idiopathic autism revealed a significant deficit of neuronal volume in 14 of 16 examined cortical regions and subcortical structures in 4- to 8-year-old autistic children, but the volume increased to the control group level in the majority of examined structures in teenagers and adults [60]. This comparative study of two autistic cohorts confirmed the hypothesis that abnormal neuronal soma growth is a common developmental defect in autism of unknown etiology and autism associated with dup(15), but also revealed that in the dup(15) autism cohort, the pattern of age-associated changes of neuronal soma is different than in idiopathic autism. The significant neuronal volume deficit detected in 11 of the 25 brain regions examined (44 %) and the uniform but insignificant deficit in another 11 regions demonstrate smaller neurons in 88 % of brain regions in 9- to 26-year-old individuals with dup(15) autism but not in idiopathic autism. The detected deficit of neuronal soma volume in teenagers and young adults with dup(15) autism appears to be a sign of a permanent defect driven by 15q11-q13 duplications.

### Study limitations

Clinical records reveal autism in 81 % and ASD in 92 % of subjects with dup(15) [12, 14]. A similar prevalence of autism (eight of nine cases; 89 %) in a postmortem-examined group does not allow determination of the difference between the pattern of pathology in subjects with dup(15) with and without autism. Examination of the brain of a female with dup(15) diagnosed with maternal origin tetrasomy [46,XX,trp(15)(q11.2q13); subject 02–09 [63], severe ID, cerebral palsy, and intractable epilepsy with the onset of seizures at the age of 9 years revealed the lowest brain weight in the examined dup(15) group [890 g; 27 % less than in dup(15) autism (1,215 g)] and five types of focal developmental abnormalities (two types of heterotopias in the hippocampus and three types of dysplasia in the dentate gyrus) similar to the type, topography and prevalence of alterations detected in eight individuals with dup(15) with autism. Stereology revealed that in the majority of examined brain structures, the volume of neuronal soma was close to the average volume detected in the dup(15) autism group. The type and topography of neuropathological changes in this case was similar to those in eight cases diagnosed with dup(15) and autism. However, severe microcephaly and the spectrum and severity of clinical alterations distinguished this case from eight other cases and hampered, but did not exclude, the diagnosis of autism. In summary, this cohort does not allow identification of the neuropathological profile of dup(15) without autism.

The second factor limiting this comparative study is the narrow age range (9–26 years) in the dup(15) group. Our previous study revealed opposite trajectories of neuronal volume changes throughout the lifespan in the idiopathic autism and control groups. The significant neuronal volume deficit detected in 14 of 16 examined brain regions in 4-to 8-year-old children with idiopathic autism decreases to four regions in the 23- to 60-year-old autistic subjects when compared to the control group. However, this shift is in part the result of a physiological decrease in neuronal volume in the majority of examined brain structures and their subdivisions in the control cohort [60]. Due to the limitation of the age range to 9–26 years in the dup(15) group, this study demonstrates a different trajectory of neuronal growth in adolescents and young adults with dup(15) autism, but neuronal volume alteration in early childhood and late adulthood cannot be determined in this cohort. Moreover, the sampling for this study does not allow for conclusions regarding the neocortex.

The next factors that may affect conclusions from this postmortem study of a cohort diagnosed with dup(15) autism is 87 % prevalence of the epilepsy, the 50 % prevalence of intractable epilepsy and the 87 % prevalence of SUDEP whereas an electronic survey of dup(15) families revealed 63 % prevalence of epilepsy [66]. One may assume that SUDEP is associated with more common and more severe developmental pathology causing intractable epilepsy, especially a high prevalence of focal developmental abnormalities. As a result, this cohort and the detected pattern of developmental abnormalities reflects pathology typical for dup(15) autism with high prevalence of epilepsy and intractable epilepsy.

This study of postmortem material and clinical records preserved in past 14 years is based on postmortem ADI-R [1] diagnostic criteria and interpretation is centered on reports published before implementation of DSM-5 [67]. The majority of comparative studies evaluating clinical cohorts according to both DSM-IV-TR and DSM-5 ASD criteria indicate that only between 50 % and 75 % of individuals will maintain ASD diagnosis when new criteria are applied [68].

### Regional abnormal neuronal growth and focal developmental abnormalities in dup(15)/autism as major factors contributing to clinical phenotype

Table 5 summarizes the similarities and differences between clinical and neuropathological findings in idiopathic autism and syndromic autism caused by dup(15). The difference between dup(15) autism and idiopathic autism is not limited to the persistent deficit of neuronal volume in teenagers and adults with syndromic autism. Focal developmental abnormalities of neuronal proliferation, dysplastic changes and defects of neuronal migration are present in almost all examined subjects in these two cohorts, but the number of focal developmental defects is several times higher in dup(15) autism cases. Moreover, the topography of focal alterations is different, with a striking concentration in the hippocampus (alveus, dentate gyrus, Ammon’s horn) found in 89 % of dup(15) autism cases, but a low prevalence (10 %) in idiopathic autism cases, and the opposite pattern in the cerebral cortex, with focal dysplastic changes in 10 % of dup(15) autism cases and in 50 % of idiopathic autism cases [46].

**Table 5:** Comparison of results of clinical and neuropathological studies of idiopathic autism and dup(15) autism

Clinical and structural alterations		Dup(15) autism	Idiopathic autism
Autism prevalence and severity	Clinical data	81 % of subjects met criteria for autism and 92 % for ASD [12, 14]. Autism diagnosed in 69 % of subjects [36]	Mild 55 %; Moderate 30 %, Severe 15 % [84]
	Postmortem study	Autism diagnosed in 89 % of children and young adults	100 %
Intellectual disability	Clinical data	5/5 severe-moderate ID [85, 86]	IQ < 70, 31 %; IQ 71–85, 23 %; IQ > 85, 46 % [84]
	Postmortem study	Profound, ID in 67 %; severe & moderate ID in 33 %	Moderate in 3 cases with tested IQ
Epilepsy	Clinical data	Epilepsy in 63 % [66]	Epilepsy in 33 % [71]. Epileptiform activity in 31 % of autistic children without epilepsy [75]
	Postmortem study	Epilepsy in 87 % including intractable epilepsy in 62 %. SUDEP in 87 % of cases	Epilepsy in 50 % of subjects SUDEP in one case/eight (12 %)
Brain weight	Postmortem study	1,215 g	1,611 g (1,376 g in control subjects)
Focal developmental defects [46]	Heterotopias (alveus, CA4, DG)	89 %	10 % (*p* < 0.001)
	DG dysplasia	89 %	10 % (*p* < 0.001)
	Cerebral cortical dysplasia	0 %	50 % (*p* < 0.03)
	Cerebellar heterotopias	56 %	60 % (ns)
	Cerebellar flocculus dysplasia	75 %	50 % (ns)
	Subependymal nodular dysplasia	22 %	10 % (ns)
Neuronal soma volume deficit (compared to control in Wegiel et al. [60] and current study)		-	In 14/16 (87 %) regions in 4 to 8 years old [60]
		11/25 (44 %) regions in 9 to 26 years old	In 3/16 (19 %) regions in 11 to 23 years old [60]
		-	In 4/16 (25 %) regions in 22 to 49 years old [60]

The concentration of focal developmental defects in the hippocampus in dup(15) autism coincides with a very uniform and significant deficit of neuronal soma volume in three sectors of the Ammons horn (CA2, 13 %; CA3,19 %; and CA4, 20 %) and an insignificant deficit in the CA1 sector. Strikingly small neurons and clustering of small neurons in CA1 and CA4 sectors are similar to the morphology of small neurons in the distinct foci of microdysgenesis detected in other parts of these sectors. This pattern suggests morphological and possibly, etiological, continuity between the diffuse pattern of abnormal growth of neurons and the focal microdysgenesis with small neurons in the dup(15) autism cohort. Similar consistency of significant diffuse deficit of neuronal volume and focal clustering of small neurons as in microdysgenesis was found in the entorhinal cortex, including islands of stellate neurons (−20 %) and pyramidal neurons in layer III (−19 %). Axons of these neurons form a perforant pathway projecting to the dentate gyrus, which demonstrates extremely prominent multiple and diverse developmental defects, including hyperconvolution of the dentate gyrus, focal thickening or thinning of granular layer, fragmentation of granular layer resulting in formation of multiple detached islands of granular neurons, duplication of the granular layer, protrusions of the granular neurons into the molecular layer and ectopias of granular neurons in the molecular layer of the dentate gyrus and in the CA4 sector [46]. The perforant pathway, composed of glutaminergic fibers, has an excitatory action on the granular neurons in the dentate gyrus and directly on neurons in the CA1, CA2 and CA3 sectors. The next link in this network is the dentate gyrus, with glutaminergic axons of granular neurons, which stimulate the dendrites of neurons in the CA3 and CA4 sectors, whereas axons of CA3 and CA4 neurons (Schaffer collaterals) reach the apical dendrites of CA1 sector [69]. These data reveal that all portions of the perforant pathways integrating structurally and functionally the entorhinal cortex and hippocampus are characterized by abnormal neuronal growth and focal microdysgenesis affecting connectivity in the limbic system. The third factor contributing to limbic system dysfunction appears to be the combination of (a) abnormal growth of neurons in the amygdala, resulting in a significant—23 %—deficit of neuronal volume detected in this study, and (b) multifocal microdysgenesis in the amygdala, with the mixture of very small neurons and abnormally large neurons reported previously [46].

These findings demonstrate the simultaneous occurrence of (a) developmental defects of neuronal growth in three major components of the limbic system, the entorhinal cortex, hippocampus and amygdala, and (b) focal developmental defects of neuronal migration and microdysgenesis in several subdivisions of the hippocampal formation and amygdala, which might be etiologically linked. The combination of both types of developmental abnormalities may contribute to the early onset of intractable seizures and SUDEP reported in the six of eight individuals (75 %) with dup(15) autism examined in this postmortem study. The absence of neuronal volume deficit in the majority of limbic system subdivisions in the age-matched idiopathic autism group, several times less often focal developmental defects, and the lower prevalence of SUDEP (in one of eight cases) in idiopathic autism suggest similar core pathology but different subpatterns in autism of unknown origin and syndromic autism caused by dup(15).

### Contribution of epilepsy to cognitive deficits and worsening autistic behavior

In autism, epilepsy is a dynamic disorder that affects brain development and function and changes clinical phenotype. After a first peak in early childhood, resulting in 12 % prevalence in the ASD cohort from 2 to 17 years of age, the prevalence increases to 26 % in children older than 13 years of age [70] and 30–39 % in cohorts that include a high number of adolescents and adults with autism [71–73]. Recently there has been increasing interest in the occurrence and role of epileptiform activity in autism in the absence of epilepsy [74]. Sleep electroencephalograms reveal epileptiform activity in 31 % of 36-month–old children diagnosed with autism [75], whereas continuous video-EEG telemetry detects epileptiform activity in 19 of 32 (59 %) children from 2 to 13 years of age diagnosed with autism without seizures. Moreover, interictal epileptiform EEG abnormalities have been detected in 11 (73 %) of the 15 subjects with autism without epilepsy [76]. Widespread neuropathological changes detected postmortem and growing evidence from functional and structural imaging may show that epileptiform activity is a common developmental abnormality that is more prevalent than clinically diagnosed epilepsy [74].

The developmental regression observed usually between 12 and 24 months of age occurs in up to 33 % of children with autism [38, 77]. Developmental regressions have been detected not only in idiopathic autism but also in 63 % of dup(15) subjects with epilepsy [66]. Epilepsy, autism and IDs may be related to a common brain pathology [78]. Individuals with both autism and more severe cognitive impairment are at higher risk of epilepsy [79]. Most studies revealed an association between low IQ and increased prevalence of epilepsy in autism [80–82]. Meta-analysis of 10 studies demonstrated a higher pooled epilepsy prevalence rate in an autistic cohort with than without ID [83]. Defects of neuronal growth in almost all examined regions in idiopathic autism [60] and in dup(15) autism with a high prevalence of epilepsy and ID might be a marker of global developmental encephalopathy and of the involvement of similar pathomechanisms contributing to autism, ID and epilepsy.

## Conclusions

Alterations of neuronal growth trajectory throughout the lifespan are a core pathology in idiopathic autism and in autism caused by dup(15). The study suggests that different topography and severity of developmental abnormalities, including increased prevalence of focal developmental abnormalities in the limbic system and prominent deficit of neuronal soma volume in the hippocampus, entorhinal cortex and amygdala may contribute to the high prevalence of IDs, the early onset of intractable epilepsy and the high rate of SUDEP in dup(15) autism.

### Consent

This postmortem study has been performed using anonymized, coded brain tissue samples. Selected clinical records were extracted from the anonymized, coded Autism Speaks Autism Tissue Program database by authorization to the project’s principal investigator.
